# Supplementary material for: Cohort profile: the Swedish Inception Cohort in inflammatory bowel disease (SIC-IBD)
Source: BMJ Open. 2025 May 6;15(5):e099218. doi: 10.1136/bmjopen-2025-099218 (PMC12056626; doi:10.1136/bmjopen-2025-099218)
Supplement: online supplemental file 1 [file bmjopen-15-5-s001.docx]

**Supplementary Table 1**

|  | **Crohn's disease n=142** | **ulcerative colitis n=201** | **IBD-unclassified n=24** |
| --- | --- | --- | --- |
| Aggressive disease, n (%) | 53 (37.3) | 48 (23.9) | 1 (4.2) |
| Indolent disease, n (%) | 83 (58.5) | 144 (71.6) | 22 (91.7) |
| Unknown*, n (%) | 6 (4.2) | 9 (4.5) | 1 (4.2) |

*Missing information about clinical outcomes due to incomplete follow-up during the first year after diagnosis.
