# Supplementary material for: Cohort profile: the Swedish Inception Cohort in inflammatory bowel disease (SIC-IBD)
Source: BMJ Open. 2025 May 6;15(5):e099218. doi: 10.1136/bmjopen-2025-099218 (PMC12056626; doi:10.1136/bmjopen-2025-099218)
Supplement: Supplementary file 2 [file bmjopen-2025-099218.reviewer_comments.pdf]

PEER REVIEW HISTORY

BMJ Open publishes all reviews undertaken for accepted manuscripts. Reviewers are asked to complete a checklist review form and are provided with free text boxes to elaborate on their assessment. These free text comments are reproduced below.

ARTICLE DETAILS

Title (Provisional)

Cohort profile: The Swedish Inception Cohort in inflammatory bowel disease (SIC-IBD)

Authors

Salomon, Benita; Grännö, Olle; Bergemalm, Daniel; Strid, Hans; Carstens, Adam; Hjortswang, Henrik; Lundstrom, Maria Ling; Hreinsson, Johann; Almer, Sven; Bresso, Franscesca; Eriksson, Carl; Grip, Olof; Blomberg, André; Marsal, Jan; Nikaein, Niloofar; Bakhtyar, Shoaib; Lindqvist, Carl Mårten; Hultgren-Hornquist, Elisabeth; Magnusson, Maria K; Keita, Åsa V; D'Amato, Mauro; Repsilber, Dirk; Öhman, Lena; Söderholm, Johan D.; Carlson, Marie; Hedin, Charlotte; Kruse, Robert; Halfvarson, Jonas

---

VERSION 1 - REVIEW

**Reviewer**	**1**
**Name**	**E M'Koma, Amosy**
**Affiliation**	**Meharry Medical College School of Medicine, Biochemistry, Cancer Biology, Neuroscience & Pharmacology**
**Date**	**17-Mar-2025**
**COI**	**None**

Inflammatory bowel diseases (IBDs), including Ulcerative Colitis (UC), Crohn's Colitis (CC), and Indeterminate Colitis (IC), remain challenging ambiguity diagnostically and prognostically with no single diagnostic gold standard available. This uncertainty in diagnosis has persisted for over 60 years, resulting in delays in appropriate treatment and suboptimal patient outcomes. There is an urgent need for noninvasive or minimally invasive biomarkers to enhance diagnostic precision, enable earlier intervention, and improve personalized care for IBD patients. The global market for IBD diagnostic and prognostic biomarkers expected to grow at a compound annual growth rate (CAGR) of 4.2% from 2023 to 2030, driven by increasing IBD prevalence globally and the demand for better diagnostic tools.

*Sweden has a tax-funded universal healthcare system for all. Therefore, attending physicians are responsible for diagnosing and managing all IBD patients. Herein Salomon et

al. is large prospective multicenter Swedish inception cohort study of inflammatory bowel disease (SIC-IBD) to establishing the diagnostic and prognostic biomarkers involving suspected IBD patients in eight-Swedish referral hospitals. Non-pediatric patients aged ≥18 years with GI symptoms referred to the GI unit between 11/2011 through 03/2021 and enrolled eligible. Reason for exclusion criteria included a prior diagnosis of CD, UC and/or IC, or inability to provide informed consent or to comply with protocol requirements. After obtaining written informed consent, all patients underwent a routine diagnostic work-up for IBD, following clinical practice. Based on the diagnostic work-up investigators established inception cohort enabling collection and generate clinical data and multi-omics datasets allowing analyses for translation into candidate biosignatures to support clinical decision-making in IBD clinical settings. Patients included in the study were classified according to the Montreal classification as (i) incident IBD, i.e., ulcerative colitis (UC), Crohn's disease (CD) and indeterminate colitis (IC, alias IBD-U) or (ii) symptomatic non-IBD as control where IBD was ruled out (a) as primary control group with other GI diseases or (b) secondary control group healthy without any GI disease. Eligible patients prospectively followed according to clinical practice, with data collected for up to 10 years for those with IBD. Furthermore, a group of healthy controls, without GI disease, included at baseline. In Table 1 it depicts the number of IBD patients analyzed as well as those with GI symptomatic and asymptomatic as controls. Details on clinical disease activity at baseline are herein provided in Table 2 and Table 3 overview of biospecimens from individuals within the SIC-IBD and easy to follow. The well-summarized number of individuals included at each hospital, the number of patients excluded and the final number of patients and controls in the SIC-IBD is herewith depicted in Fig. 1 and the managerial/operational organization of BIO IBD shown in Fig. 2. The database updated every 6-12 months, including follow-up visits and data freeze created at each update.

I find this SIC-IBD, largest cohort and one of few large-scale IBD cohorts of adult patients with integrated biobanking interesting and informative with caution that evaluations based on these cohorts are likely to overestimate the diagnostic capacity of potential biosignatures. Uniquely, this study has included patients with symptoms indicative of IBD without any signs of the disease at diagnostic work-up or during follow-up. Thus, both cases with IBD and symptomatic controls represented an unselected sample of patients referred to secondary care for the suspicion of IBD. Healthy controls as a second control group to gain insight into the etiology of IBD and to characterize pathways related to disease pathogenesis. This is a well written/summarized study.

Reviewer **2**

Name **Frigstad, Svein Oskar**

Affiliation **Vestre Viken Hospital Trust, Department of Medicine**

Date **23-Mar-2025**

COI **None**

A balanced and informative presentation of a well-established cohort. It is nicely described how the cohort may answer an unmet need in the research within the field of markers that may be useful for establishing diagnosis and prognosis in IBD. I have a few minor comments that may be addressed in a revision of the manuscript.

It is stated that all patients are newly diagnosed. This is plausible as patients are included on refrral to a specialist center. Nevertheless preliminary results suggest that up to 10% had some form of treatment for IBD before inclusion. It would benefit the paper to comment on this. Maybe some patients were evaluated in primary care or a clinic (private or public) that initiated some form of basic treatment. Also 'newly diagnosed' may be mentioned in the abstract.

In the Cohort description on page 6 the included hospitals are listed. I would like to know the catchment area of each hospital (or at least a total number) as it is stated that most patients will have follow-up in a specialist care center and this number may elucidate the incidence even if patients not included are not presented. The Swedish health care system is solid, and it may be assumed that the majority of patients presenting symptoms are referred to a hospital for follow-up.

I would like some comments and a discussion on the criteria for defining an aggressive disease course (presented in table 4). They seem sensible, but here it would strengthen the paper to offer some thoughts on criteria used in other cohorts and why these are chosen as a composite outcome measure, also please add references.

The references given as 37 and 38 are poster presentations. That is fine, but it should be stated also for reference 37 that these are preliminary data (the same way the authors have done for reference 38). If a final paper has been published, the reference may of course be updated.

Lastly, any further comments on why the fecal sampling were that much lower that blood tests. Any learning points for other researchers. Otherwise the study, project organisation and options for collaboration is elegantly described.

VERSION 1 - AUTHOR RESPONSE

__Reviewer 1:__

Dr. Amosy E M'Koma, Meharry Medical College School of Medicine, Vanderbilt University Medical Center

Comments to the Author: [...] I find this SIC-IBD, largest cohort and one of few large-scale IBD cohorts of adult patients with integrated biobanking interesting and informative with caution that evaluations based on these cohorts are likely to overestimate the diagnostic capacity of potential biosignatures. Uniquely, this study has included patients with symptoms indicative of IBD without any signs of the disease at diagnostic work-up or during follow-up. Thus, both cases with IBD and symptomatic controls represented an unselected sample of patients referred to secondary care for the suspicion of IBD. Healthy controls as a second control group to gain insight into the etiology of IBD and to characterize pathways related to disease pathogenesis. This is a well written/summarized study.

Reply: We thank Dr. Amosy E M'Koma for the positive and encouraging feedback.

__Reviewer 2:__

Dr. Svein Oskar Frigstad, Vestre Viken Hospital Trust

Comment to the Author: A balanced and informative presentation of a well-established cohort. It is nicely described how the cohort may answer an unmet need in the research within the field of markers that may be useful for establishing diagnosis and prognosis in IBD. I have a few minor comments that may be addressed in a revision of the manuscript.

Reply: We thank Dr. Svein Oskar Frigstad for the positive feedback and his important comments.

Comment 1: It is stated that all patients are newly diagnosed. This is plausible as patients are included on refrral to a specialist center. Nevertheless preliminary results suggest that up to 10% had some form of treatment for IBD before inclusion. It would benefit the paper to comment on this. Maybe some patients were evaluated in primary care or a clinic (private or public) that initiated some form of basic treatment. Also 'newly diagnosed' may be mentioned in the abstract.

Reply: Thank you for the insightful comment. It is true that about 9% of patients had started treatment before inclusion. In about half of these cases, the patients had received only one or a few doses of corticosteroids in the days before inclusion. We have now clarified this in the manuscript by adding a sentence discussing this aspect within the limitations of the cohort (**p.21; l.411-414**).

"Furthermore, not all included patients were treatment-naïve, which could influence the molecular data and may warrant sensitivity analyses. Approximately 9% of patients had initiated treatment before inclusion, most commonly with one or a few doses of corticosteroids in the days preceding inclusion"

Comment 2: In the Cohort description on page 6 the included hospitals are listed. I would like to know the catchment area of each hospital (or at least a total number) as it is stated that most patients will have follow-up in a specialist care center and this number may elucidate the incidence even if patients not included are not presented. The Swedish health care system is solid, and it may be assumed that the majority of patients presenting symptoms are referred to a hospital for follow-up.

Reply: Unfortunately, estimating the total catchment area for all hospitals is challenging, as only a few have had well-defined and stable catchment areas over time. In Stockholm, patients were recruited at the Karolinska University Hospital in Solna and Huddinge and also at Ersta Hospital. However, referral patterns from primary care to these hospitals have not been stable over time, due to changes in healthcare organisation. Similarly, patients were recruited at the University Hospitals in Lund and Malmö, where major structural changes in health care organisation occurred during the study period.

Despite these limitations, we believe most patients with suspected IBD are referred to secondary care for diagnostic workup. However, we acknowledge that this cohort is not strictly population-based, and that selection bias may have influenced study population. We have clarified this aspect in the revised Discussion section **(p. 20 l. 400-402).**

"The absence of a population-based cohort design could be a limiting factor when interpreting associations between exposures and clinical outcomes within SIC-IBD, as selection bias may have been introduced."

Comment 3: I would like some comments and a discussion on the criteria for defining an aggressive disease course (presented in table 4). They seem sensible, but here it would strengthen the paper to offer some thoughts on criteria used in other cohorts and why these are chosen as a composite outcome measure, also please add references.

Reply: Thank you for the comment. As the Montreal classification does not include a category for disease outcome, and no universally accepted gold standard exists to define disease course in IBD, we have used a composite outcome measure for Crohn's disease and ulcerative colitis. Similar criteria have been applied in the REACT and REACT-2 trials for Crohn's disease, and comparable event-based definitions have been used in the IBSEN III cohort to evaluate disease course. We have expanded the discussion on this topic in the revised manuscript and added additional supporting references (**p.20; l. 381-396**)

> *"A composite outcome was chosen to categorise disease course since no established gold standard for defining or classifying IBD course currently exists. The Montreal classification provides a framework for Crohn's disease location and behaviour, and extent of ulcerative colitis but does not capture disease progression over time [23]. Several other cohorts, including the European Crohn's and Colitis Organization's Epidemiological Committee (EpiCom) inception cohort [14] and Inflammatory bowel disease in South-Eastern Norway cohort III (IBSEN III) [41], have recorded disease-related events such as IBD-related surgery, disease complications, and hospital admissions to assess disease severity over time. Similarly, the Randomised Evaluation of an Algorithm for Crohn's Treatment (REACT) [19] and REACT-2 [39] trials employed a composite outcome to define disease course in Crohn's disease using similar criteria. While a single criterion, such as treatment escalation or new disease complications in Crohn's disease [44,45], may facilitate interpretability, a composite outcome allows a more comprehensive assessment of disease severity over time. This approach captures multiple dimensions, including difficulties in controlling inflammation, fibrosis-related complications, and poor treatment response, thereby offering a broader perspective on disease course.*

Comment 4: The references given as 37 and 38 are poster presentations. That is fine, but it should be stated also for reference 37 that these are preliminary data (the same way the authors have done for reference 38). If a final paper has been published, the reference may of course be updated.

Reply: Thank you for pointing out this inconsistency. The final paper has been accepted for publication but has not yet been published. We have now clarified this in the manuscript **(References, p.29 l.606).**

> *"37 Pertsinidou E, Salomon, B, Bergemalm D, et al. Anti-integrin αvβ6 IgG antibody as a diagnostic and prognostic marker in ulcerative colitis: A cross-sectional and longitudinal study defining a specific disease phenotype. (Accepted for publication in J Crohns and Colitis on March 14, 2025)"*

Comment 5: Lastly, any further comments on why the fecal sampling were that much lower that blood tests. Any learning points for other researchers. Otherwise the study, project organisation and options for collaboration is elegantly described.

Reply: We sincerely thank the reviewer for the question regarding the sample numbers, which led us to detect a mistake in the reported number of faecal samples. We have now corrected the error and inserted an updated version of **Table 3 (p.13)** below. The correct number of faecal samples is higher than originally reported but remains significantly lower than the number of blood samples. We did not conduct additional analyses or follow-up questionnaires that could provide further insights into the reasons for this discrepancy. However, we have highlighted the challenge of collecting faecal samples in the revised manuscript **(p.5; l.116-118).**

> *"However, the diagnostic accuracy of CRP is too low for reliably identifying IBD patients, and the utility of FCP is hampered by poor patient adherence to faecal sampling [8,9]."*

Notably, the collected faecal samples are intended for molecular analyses of additional faecal markers and microbiome analyses. However, some patients provided a sample for the assessment of faecal calprotectin in clinical routine, without providing extra samples for research purposes. We have now clarified this in the text of **Table 3 (p.13)**. Additionally, faecal calprotectin data were missing for approximately 36% of individuals. The proportion of missing values was comparable across patient groups, indicating that missing data were not specific to a particular group (Crohn's disease, 37%; ulcerative colitis, 34%; IBD-U, 25%; symptomatic controls, 40%; and healthy controls, 32%).

Table 3. An overview of biospecimens collected at baseline from individuals within the Swedish Inception Cohort in inflammatory bowel disease (SIC-IBD)

	**Crohn's Disease n=142**	**Ulcerative colitis n=201**	**IBD-unclassified n=24**	**Symptomatic control n=168**	**Healthy control n=59**
**Serum, n (%)**	137 (96)	199 (99)	23 (96)	164 (98)	59 (100)
**Faecal, n (%)***	99 (70)	137 (68)	20 (83)	112 (67)	56 (95)
**Intestinal biopsies, n (%)**	92 (65)	161 (80)	18 (75)	155 (92)	58 (98)
**Urine, n (%)**	62 (44)	91 (45)	9 (38)	90 (54)	58 (98)

**Faecal samples for microbiome analyses and for additional faecal marker. Samples for faecal calprotectin were handled separately.*
IBD, inflammatory bowel disease
